# The extraordinary makes the ordinary visible - nursing students’ experiences of their learning in clinical practice during COVID-19: a qualitative study

**DOI:** 10.1186/s12909-022-03796-8

**Published:** 2022-10-25

**Authors:** Lena Engqvist Boman, Åsa Johansson Stark, Carina Georg, Charlotte Silén

**Affiliations:** 1 Department of Neurobiology, Care Sciences and Society, Karolinska Institutet, Stockholm. Postal address: Division of Nursing, Alfred Nobels Allé 23, C3, SE-141 83 Huddinge, Stockholm, Sweden; 2 Department of Learning, Informatics, Management and Ethics (LIME) Karolinska Institutet, Solna, Stockholm, Sweden

**Keywords:** Nursing students, Learning, COVID-19, Clinical education, Focus groups

## Abstract

**Background:**

The COVID-19 crisis had a significant impact on health care and nursing education as a large part of it is carried out in clinical practice. However, it is not known how the learning situations during the pandemic affected students’ learning. To deepen the understanding of students’ learning, learning theories within a constructive paradigm is used as a framework for this study. The purpose of the study was to explore nursing students´ perceptions of their learning in clinical practice during COVID-19.

**Methods:**

In this interpretative qualitative study, seven focus group discussions were conducted with 21 nursing students at different stages of the nursing programme, all of whom performed clinical practice during the outbreak of COVID-19. The analysis of the discussions was performed with interpretative content analysis related to theoretical assumptions about learning.

**Results:**

The learning situation was characterised by chaos and confusion affecting both the students’ opportunities to learn and what they learned. Despite the uncertainty the students appreciated having experienced this unique situation, which contributed to valuable learning. Things otherwise taken for granted or not encountered before became visible. The learning processes were characterised by complexity and challenges that hindered or stimulated learning. It depended on the student’s approach and the management of the clinical education. Concerns about one´s own and relatives’ health, and not being able to finish studies, also affected learning. The students learned about important measures during a pandemic regarding hygiene, care organisation, communication, and the multifaceted role of the nurse.

**Conclusion:**

Unpredictable situations such as a pandemic can lead to unique learning since “the extraordinary makes the ordinary visible”. The students learned things additional to the formal learning outcomes, and the experiences strengthened their will to become nurses. Challenges due to a crisis can become important driving forces for learning, if not experienced as overwhelming. Some students felt they received space for own initiatives and responsibility while others felt lost and abandoned. Preparing for a crisis means preparing for an unknown future. Students therefore need to experience dilemmas and uncertain situations and reflect in a safe environment.

## Background

The COVID-19 outbreak in 2020 soon turned into a crisis marked by uncertainty, existential threats, and urgent action [1]. The uncertainty during the COVID-19 crisis concerned the lack of knowledge about the virus and its treatment. The existential threat was about human health and life, which led to urgent measures to reduce the effect of the virus [2]. The pandemic had a profound effect on health- and elderly care [3, 4]. Several wards were transformed into care for patients with COVID-19, which led to an increased workload and health care staff and students being infected or afraid of becoming so [5, 6]. This was a unique situation that led to consequences for the nursing students’ clinical practice and offered rich opportunities for learning, but also great challenges and experiences of uncertainty [7–9]. Clinical practice is important for students’ learning and their development of professional competence as it allows them to have direct contact with patients and practitioners in their own profession and that of others [10]. When students come to their clinical placement, they encounter complex social and organisational contexts, which they must understand and gain acceptance into in order to learn [11]. Clinical practice is characterised by being concrete, visual, and tangible [12]. People and things are concretely available, which enables the sense of touch to be used to obtain further information about what one observes, hears, and smells. All these stimuli evoke thoughts, feelings, and a desire to understand and be able to act. Meeting severely ill and dying patients can evoke strong emotions that engage the students; thus, they might need support [13]. A literature review shed light on the importance of supervisors’ support during clinical practice to enhance learning [14]. During a crisis, as the COVID-19 pandemic, the supervisor also needs to address students’ coping strategies to handle stress and anxiety, as reported in several studies [15–20].

Learning theories within a constructivist paradigm is the framework for this study [21–25]. The theories highlight different aspects of learning but share some basic assumptions. These are that learning processes are active constructive processes in the learner, and information is processed cognitively, emotionally and through trying and practical actions. A basic driving force in the individual is to try to understand and handle situations that are perceived as relevant and meaningful. Understanding and attitudes, self-perception and skills are constructed by the individual in interaction with others and the environment. Learning means experiencing and taking in stimuli with all of one’s senses, which are then processed and internalised, becoming an integral part of the individual. The uncertain situation caused by COVID-19, as described above, can be assumed to have affected the learning situations and thus the students’ learning. Several studies have reported about the effect on students’ emotions and how it affected their learning during COVID-19 [15–20]. However, there is a lack of studies about how the students’ learning can be understood in relation to what we know about learning in general. Therefore, this study aims to explore nursing students´ perceptions of what and how they learned in clinical practice during COVID-19 and relate it to explanations based on learning theories.

## Methods

An interpretative qualitative approach was used in the study to explore nursing students’ perceptions of learning [26].

### Setting

This study was conducted at a medical university in Sweden, where the nursing programme is a three-year post-secondary education leading to a license to practice as a registered nurse and a Bachelor of Science degree in nursing. The nursing programme consists of both theoretical and clinical courses. Approximately half of the education is dedicated to clinical education. In the clinical education students learn through experience with actual patients and get opportunity to develop professional competencies under the supervision of a registered nurse. The clinical education is carried out in a variety of clinical settings as hospitals, community sites, patients’ homes, and primary care.

In Sweden most nursing students remained in clinical practice during the pandemic. Provided that the students received adequate instructions, they followed the same guidelines as healthcare workers. This meant that most students completed all or part of their clinical placement during the first wave of the pandemic, and some students were directly involved in the care of patients with COVID-19. A risk assessment was performed before the students entered the clinical settings with the aim to protect students and fragile patients, and to reduce the spread of infection. For example, students who were pregnant or had a compromised immune system did not attend clinical practice.

### Participants

All nursing students (n = 735) from semesters (S) 2, 3, 4, 5 and 6 had clinical practice in the spring of 2020 and were invited to participate in the study. A purposive sampling was done by recruiting students from different levels of the nursing programme. The purpose was to collect rich data based on the varying experiences of students related to their different clinical placements and their level of knowledge. [26]. The invitation was sent by e-mail in connection with the course evaluation at the end of spring 2020. Twenty-one (16 women and five men) accepted. The participants met patients with or without COVID-19 at different types of placements. The placements correspond with those in the syllabus of the nursing programme (Table 1).

**Table 1:** Participants and clinical placements

	Semester 2	Semester 3	Semester 4	Semester 5	Semester 6	Total
**Number of participants**	8	5	2	3	3	21
**Clinical placements**	Nursing homes	Hospital wards	Primary care Psychiatric ward	Hospital ward Interprofessional education ward Outpatient department	Geriatric wards	

### Data collection

Seven focus group discussions were performed in October 2020, with 2–5 participants per group. Each group had students from the same semester, as they shared experiences that might facilitate discussions [27]. Due to the COVID-19, online data collection via a video conferencing platform was an appropriate method to engage students without spreading the infection. Three to five participants are recommended to facilitate moderation and interaction in the virtual environment [28]. All authors, two at a time, took part in the focus groups. One initiated the discussion, and the other observed the non-verbal and verbal interaction, and supplemented with questions for clarification and in-depth understanding. The students were familiar with the technology which facilitated participation in the focus group discussions. The discussions started with one open question, which was phrased “Tell us about your clinical practice during COVID-19 – when you think back, what is your strongest impression?” Domains in focus for further discussions were based on the theoretical framework and concerned students’ experiences of learning, supervision, support, preparation, reception, opportunities to be involved in patient care, and perceptions related to COVID-19. The focus group discussions lasted between 70 and 80 min and were audio recorded and transcribed verbatim by professional transcribers not involved in the research. Two of the authors had had a previous teaching relationship with some of the students but none had any position of dependency with the authors during the time of data collection. In addition to established ethical principles, certain aspects must be addressed in online research regarding privacy, confidentiality, and data security [29]. Privacy was assured through personal invitations to the online conference and the host controlled who entered the meeting. The control of the recordings was limited to the host. To ensure confidentiality of data the participants were given a number which was referred to during the recording instead of the participants’ names. The video recordings were deleted immediately after the focus group discussions and only the audio recordings were stored at the host’s computer and used in the handling of data. The students were informed about the procedure before the recordings and were asked to evaluate, orally or in writing, their participation at the end of the focus group discussions. All appreciated the discussions, and none wanted to withdraw their participation.

### Data analysis

The analysis of the transcriptions was performed with an interpretative content analysis of both the manifest and latent content [30, 31]. The analysis followed an iterative process of going back and forth between parts and wholes of the data and addressing theoretical assumptions about learning within the constructivist paradigm. This procedure is inspired by the abductive analysis [32] aimed to shed light on variations in the students’ perceptions related to learning theories. The analysis of the manifest content, i.e., what was said in the focus group discussions, was followed by an interpretation of the latent content due to this procedure:

- Each audio recording and transcript was reviewed several times and summarised in writing to get an overall picture;
- Text related to the research focus was extracted, condensed into meaning units, and coded;
- The codes were compared for differences and similarities and grouped into categories;
- The categories were interpreted as themes and sub-themes.

All authors who are experienced teachers and researchers, were involved in the analysis; each transcript was coded and categorised by two authors. The codes and categories were thoroughly discussed, revised, and agreed upon by all authors. Two of the authors interpreted the content of the categories and formed themes and sub-themes answering the questions concerning how learning was expressed and perceived and what was learnt. The themes and subthemes were further negotiated between all authors. Trustworthiness was sought by the systematic and reflexive analysis described above. All authors contributed to different perspectives and ensured increased credibility. The subthemes are presented with quotes to illustrate the interpretations of the participants’ experiences. To further strengthen the trustworthiness of the analysis, an experienced researcher outside the research group read and commented on the results [31].

## Results

In the analysis distinctive features of the students’ learning situation and how and what they learned were identified and presented in three themes and subthemes (Table 2). The quotations illustrate the interpretations of the subthemes, and the respondents are presented by number of semester (S) and participant.

**Table 2:** Students’ learning. Themes and subthemes describe the learning situation, and how and what the students learned

**Themes**	1. A chaotic and confusing learning situation	
	2. Learning processes characterised by challenges (how)	3. Increased awareness of important measures during a pandemic (what)
**Subthemes**	2.1 Learning by being part of a crisis	3.1 The extreme importance of compliance to hygienic measures
	2.2 Depending on crisis management for learning	3.2 The significance of the care organisation and work environment
		3.3 A multifaceted competence of the nurse came to the fore
		3.4 Communication measures as vital in crises and caring

### 1. A chaotic and confusing learning situation

This is an overarching theme as the learning situation affected what and how the students learned. Learning was related to the situation, characterised by uncertainty and worry during the outbreak of the pandemic. Students described the situation as unique and chaotic, and the uncertainty concerned themselves, their relatives, the health care staff, the care of patients, the organisation of the health care and the surrounding society. The extreme situation with many deaths caused by the COVID-19 also affected the students who felt that this was for real.

“They had to build two refrigerated containers just outside the mortuary. It was a bit nasty because you got so close to it and saw how many people that actually died.” (S4:15)

The uncertainty was higher in the beginning of the pandemic and depended on where the students performed their clinical practice. The themes 2.*Learning processes characterised by challenges* and 3.*Increased awareness of important measures during a pandemic* were related to these different learning situations.

### 2. Learning processes characterised by challenges (how)

Challenges, due to the chaotic situation, stimulated the learning process or became a hindrance as described in the subthemes.

#### 2.1 Learning by being part of a crisis

The students experienced the situation from the inside; they were actually involved in the overwhelming situation, which also affected them as individuals. The students’ own reactions and approaches to different events influenced their possibilities to learn from the confusing and threatening situation. It triggered students’ attention to things otherwise taken for granted or not encountered before.

“When things keep going, you may have a feeling that things work out well. But when you meet a challenge, you realise ‘Oh, no it doesn’t work’.” (S2:2)

The students described what they had noticed in relation to different levels of the health care system, their specific clinical placement, and the situation for staff, patients, and relatives. Their observations and possibilities to reflect on this situation became important and supported their learning processes. The unique and chaotic situation meant that students were given space to take their own initiatives and plan how they could benefit in their learning processes. Some students took this chance and found ways to learn by arranging how they could get supervision from nurses or other staff. When feeling neglected at one place they deliberately moved to another ward and connected to a supervisor they could trust. However, this sudden open space had almost the opposite consequences for other students. The uncertainty in the clinic regarding how to handle the situation and their own worries about getting sick hindered them from taking part in the caring and learning. The lack of nurses and sudden changes when a designated supervisor moved to another ward or became sick impacted what tasks the students became involved in. The opportunity to act as a nurse and be trusted in taking responsibility for the care of patients, benefitted their understanding of the nursing profession.

“We became kind of colleagues and the nurse said that we should be kind of responsible for certain areas. You had to learn a lot in a short time because they had no other resources.” (S2:6)

The uncertain situation meant no one was an absolute expert who could tell others what should be done or what was the best way to solve problems. This benefitted students’ learning since they became involved on equal terms in discussions about how difficulties could be handled. It contributed to their self-confidence and encouraged them to take responsibility for the care of patients and thus learn more. If the responsibility was experienced as overwhelming, the students felt abandoned and found learning difficult. Rather than learning how to take care of patients, they started to doubt their own ability.

“But we were not prepared to.…suddenly do everything completely independently the second day….and to receive patients, you feel like an idiot, and don’t know the routines.” (S5:19)

#### 2.2 Depending on crisis management for learning

The students’ own reactions and approaches interacted with the way they were treated and how the situation was handled at the clinic and the university. Experiencing support from supervisors, teachers and peers and having access to patient care facilitated the learning process. The planning for and reception of students in the clinic was affected at all clinical placements. Students’ involvement in the care and clinical work was important for their learning. To avoid being infected, students at some placements were not allowed to take direct care of patients with COVID-19. If no explicit plan to compensate for the absent encounters existed, this was a great hindrance to their learning processes. Another obstacle was the lack of protective clothing and protective equipment. This meant that students were not given priority to receive it and therefore could not be involved in patient care.

“I just felt that they did not prioritise us…. they (staff at the clinic) e-mailed corona updates to all (employees) but not to us students. We were very outside and could not keep up with all the changes. We were not allowed to do almost anything due to lack of material.” (S3:11)

When hygienic measures were not well followed it added to the students’ anxiety, as did their fear of infecting vulnerable relatives. It also affected their choices of involvement in the patient care.

“Hand alcohol disappeared, gloves disappeared, everything disappeared from all departments. We were without it for several days. It didn’t feel good at all. I refused to do anything.” (S3:11)

When the staff was able to handle their own worries, they could provide support and plan learning activities for the students. In some clinical placements, where the ordinary caring was altered, the students were well cared for by being engaged in learning through alternative tasks to practice and reflect on. When the staff was very worried and uncertain, and the lack of personnel was overwhelming, there were almost no plans for how to take care of the students. A lack of or unclear and ambiguous information from the university about possibilities to carry out the clinical practice during the pandemic, and how to deal with sick leave, added to the students’ worries. The students in their last semester worried about whether they could finish their studies. Students’ experiences of support from the university ranged from satisfactory to unsatisfactory.

Working with peers in these special circumstances was emphasised by the students as very important and beneficial to their learning. They could plan and reflect together and support each other when there was less time for the staff to pay attention to them. However, in these chaotic times, the importance of creating opportunities for students to support each other was not always noticed. Sometimes the students arranged peer learning themselves.

“I think you get a lot of help from being two students, then you can accept almost anything, as long as you are not completely alone in the situation. It doesn’t have to be simple or fun or anything like that, I just want to learn.” (S2:7)

Gaining access to encounters with patients in this unique situation promoted the students’ learning in many ways. They experienced what it was like to be dressed in protective clothing and what it meant in terms of disturbance in communicating with patients. They found it frustrating to communicate only with their voice and lose the possibility of facial expressions to show engagement and compassion.

“To touch someone with gloves, to pat someone on the cheek, who is sick and may die soon, with gloves on, it almost feels …it feels better not to do it.” (S2:3)

The students’ observations of the pandemic’s impact on the patients’ suffering and need of care made the students reflect on how they would act themselves in their future profession.

### 3. Increased awareness of important measures during a pandemic (what)

This theme describes the content of what the students learned, i.e., their increased awareness about important measures during a pandemic. The content of learning is described in the subthemes.

#### 3.1 The extreme importance of compliance to hygienic measures

The students became aware of the importance of following hygiene routines and using protective equipment to prevent virus transmission both to the patients, themselves, and their relatives. The frequent practice of careful hand hygiene and the use of protective clothes, face masks and visors entailed an extra workload but became routine for students involved in the care of infected patients. The students noticed when hygienic routines were not followed.

“They (the cleaning staff) did not change water... the mop… rags... gloves... aprons… they basically distributed the infectious agents fraternally across the ward.” (S3:10)

#### 3.2 The significance of care organisation and work environment

The students became aware of the essentials of collaboration in the team and a secure and cohesive work environment in a chaotic situation like the pandemic. Several challenges were noted among the staff, e.g., to suddenly care for patients with COVID-19 instead of the ordinary patients, difficulty adapting to the changes, conflicts over resources, and the lack of rest and emotional support. The heavy workload sometimes made the students avoid disturbing the staff, and sometimes they felt they were being used as a resource for the staff. These experiences revealed difficulties to supervise and prioritise student learning in a care organisation.

#### 3.3 A multifaceted competence of the nurse came to the fore

The role of the nurse became much clearer, namely that it is a demanding and multifaceted task. It requires considerable knowledge, flexibility, and continuous learning. The perception of the role was positive, making the students proud and motivated for further studies as well as strengthening their choice of profession.

“It is a very important role, it includes much more than what is given by the first impression…in fact, one should carry one’s professional role with pride, because we must know a lot about many things.” (S2:2)

The nurses’ exposed position in taking care of patients infected by the virus was noted, including that it is important that you take good care of yourself. The importance of presence and clarity in the nurse’s leadership for good communication between staff and patients became clear. For some students the clinical practice also contributed to increased self-confidence.

“After the clinical practice I really feel that ‘If I can handle this then I can handle anything’.” (S2:6).

#### 3.4 Communication measures as vital in crises and caring

The significance of information and knowledge among staff, and patients’ vulnerability in being isolated, was highlighted. Communication, personal meetings, clear leadership, and knowledge proved to be important measures to avoid fear, rumours, insecurity, and conflicts among staff. The students also realised the difficulties of informing and disseminating knowledge-in this case about the COVID-19-among staff and reflected on how it could be done.

“There are so many different theories at the same time, therefore we need to distribute the information simultaneously and to everybody.” (S2:3)

The students’ learning about the effect of the pandemic on patient care mainly concerned the need for personal encounters and verbal and non-verbal communication. The nurse’s pedagogical skills became clearer, as one student put it.

“I need to understand that (the information) and be able to put it in a context the patient understands and at the level the patient is interested in understanding. I think it has become clearer now during the corona.” (S4:14).

The personal encounters were emphasised, especially for elderly patients and those with dementia who were isolated for long periods of time. Some students experienced a close relationship with the vulnerable patients, which increased the students’ self-awareness. The patients were not allowed to meet relatives, and the health care staff spent as short period of time as possible with the patient to avoid the risk of being infected. Sometimes the student took the initiative to introduce a digital tool to facilitate communication between patients and their relatives as the staff lacked the knowledge about these tools. The patients’ isolation had a negative effect on their well-being according to the students. They felt sad and thought the treatment, especially when patients were left alone at the end of life, was tragic and unworthy, and this provided new insights. The care of the dead body of a patient infected with COVID-19 was perceived as a materialistic procedure rather than an emotional and respectful farewell. This observation made the students aware of the discrepancy between what they had learnt about respectful treatment and how it was applied in the care of patients with COVID-19.

“Is this really (care) with dignity? Is this the quality you can expect especially in a country like Sweden?” (S6:18)

It also became clear to the students how the protective clothing and equipment prohibited contact and communication with patients who sometimes became scared and confused. Using a face mask and visor made the facial expression invisible and affected the student’s voice. The equipment could change the student’s perceived personality, making them more like a robot among others as all staff looked the same.

“Being dressed like this means that we lose our personality. We all look the same… it became like an impersonal care… I think about those who reacted very strongly to our costumes and thought it was very scary because it looked like an apocalyptic movie where all were dressed as cosmonauts.” (S2:1)

It was hard for the students when they did not know how patients with dementia perceived the situation. The students reported different ways of managing the situation such as showing their will to help the patients, introducing themselves every time they met the patient, talking loudly, and communicating both verbally and non-verbally. An important learning experience concerned the significance of using body language and being creative to make contact and provide comfort and hope.

“I hoped it was noticed that I smiled with my eyes.” (S6:17).

## Discussion

The present study shows that despite confusion and uncertainty, the students appreciated being part of the unique situation and found it valuable for their learning. The chaotic and confusing situation affected both their possibilities to learn and what they learned. This is hardly surprising, and other studies that follow up on students’ situations during the pandemic confirm these findings [15–20]. However, this study illuminates that learning processes during this unknown and insecure time turned out to be signified by complexity. The students’ learning outcomes seemed to depend on several different interacting factors. The students’ knowledge, personality, behaviour, and reactions in the situation interacted with, e.g., how they were treated by the staff and the management of the pandemic situation on different organisational levels. Due to this complexity, there are no simple answers concerning how to deal with students’ clinical education during a crisis. To deepen the understanding of such a complexity and support transferability to other contexts, we will discuss the results in relation to theoretical assumptions and experiences about learning.

An interesting observation that permeates the results is that the extraordinary situation caused by the pandemic made the ordinary visible, for example, the importance of following hygiene routines. The students also drew attention to questions about the work environment, and the nurse’s multifaceted competence was observed and appreciated. From a learning perspective these findings can be understood based on the variation theory described by Marton [33]. A fundamental assumption is that learning includes the discernment of crucial factors in a situation and reorganising and combining them into comprehensible wholes. The basis to discern something is variation, to experience differences and figure-ground structures [34]. Thus, the extraordinary conditions the students met and had to deal with presented meaningful experiences of variations, which made them open their eyes for things to learn that they had otherwise not thought of. Adding to the meaningfulness was the students’ learning through bodily experiences. Wearing the protective and uncomfortable equipment made them aware of how it feels and how it affects contact and communication with patients, both verbally and non-verbally. Barisone et al. [35] found that, although it was demanding to be involved, it provided learning opportunities such as the importance of non-verbal communication and questions about dignity.

Using all senses and bodily experiences are important aspects of the learning process as consciousness is shaped through the body [12, 36]. This emphasises the importance of giving students the opportunity to take responsibility and be involved “for real” in the care of patients with COVID-19 and not only observe. Being involved for real seemed to have contributed to both external and internal authenticity [37–39]. The students could use all senses to process information and learn since they were part of an external context being concrete and tangible. Experiencing that their own actions had an impact on the clinical work contributed to internal authenticity. These findings raise questions concerning how to create learning spaces offering such opportunities for meaningful learning that do not include a serious event such as the pandemic. It would be possible to encourage students to take a critical approach and pay attention and reflect on common daily routines. Another way to use lessons learned is to ask the students to consider and report what they learn by using different senses in encounters with patients and being in the clinic reality. This study also shows that students learn things in addition to the formal learning outcomes. For example, being a part of a care organisation during a crisis and to perceive the impaired communication with patients when wearing protective equipment offered a shift in perspective. This highlights how learners interpret their performance in the workplace in terms of what is relevant information for their learning [40]. Therefore, performance relevant information needs to be addressed by supervisors, teachers, and in curriculum development.

The uncertain and confused situation created by the pandemic caused challenges for the students’ learning. It is striking that students describe both benefits and hindrances related to the challenges they had to face. Some students point out that they received more space to take their own initiatives and learned from the possibilities of being responsible for and practicing nursing tasks. Other students felt abandoned, and they experienced and felt that they missed learning possibilities. Experiences of challenges are known as an important driving force in learning [23, 41, 42]. However, for challenges to become a positive driving force they cannot be overwhelming or too many at a time. Students will need practice and support to develop self-confidence and strategies to cope with success as well as failure when they dare to take on challenges to learn [23, 43].

It is obvious that the students became vulnerable in different ways in their clinical practice during the pandemic. They worried about their own health as well as that of their relatives but also about not being able to finish their studies. This is a great concern for both the health care units responsible for students’ clinical practice and the university. A need for more psychosocial support, regular contact with the clinical supervisor, recognition of the difficult work situation and more space to unwind have been emphasised [33]. However, it requires a safe working environment for the supervisors as nurses have reported that insufficient institutional support during the COVID-19 pandemic caused stress [44]. None of the organisations in the present study seem to have been prepared to handle the learning situation for the students in the emerging crisis. Hopefully, the results in this study can be of help to ensure they are better prepared in the future. Barisone et al. [35] suggest that educational activities could be designed for students to process their experiences, including stress management. We would also argue that it will not be possible to be fully prepared for a crisis such as this pandemic, as the unknown is what creates a crisis [1]. Would it be possible to prepare students to better cope with the unknown and uncertainty? Barnett [45] suggests that preparing for uncertainty and the unknown is not primarily a question of epistemology, acquiring knowledge and skills, but an ontological challenge, fostering human qualities and dispositions. He claims that preparing students for the unknown will require learning spaces to practice carefulness, thoughtfulness, humility, criticality, receptiveness, resilience, courage, and stillness. It is interesting to notice that the students in the current study describe experiences and personal approaches related to the meaning of these human qualities. Introducing authentic ontological challenges to the students to cope with themselves and balance their insecurities with guidance may be one way to develop readiness for the unknown. This could be done in a safe environment by using simulation or role play creating dilemmas and uncertainty to be followed up by a facilitator who also focuses on students’ thoughts and feelings [23, 45].

It is well-known that the “reality” in health care, such as patients’ needs and a lack of resources, often precedes the task of creating good conditions for and supporting students’ learning [14]. Clinical supervisors bring to the fore the challenges of balancing patient care and facilitating student learning [46]. Students have reported that the psychosocial support and support from clinical supervisors is crucial in such chaotic situations [47]. However, despite this support, some students in the same study perceived practical worries, fewer learning opportunities and had fundamental doubts about their choice to become a nurse. Nursing students with higher anxiety levels were found to have less positive attitudes toward the nursing profession during COVID-19 [17]. No one in the present study, however, doubted their career choice. On the contrary, they were proud to become a nurse. Nevertheless, they realised how complex and demanding the professional role was and that the nurse had a vulnerable position during a pandemic. The need for supporting the students to process their experiences and prevent drop-out is stressed by Barisone et al. [35]. The students in the present study reported both good and less good support both from clinical supervisors and teachers at the university. A close collaboration between the clinical placements and the university is needed to organise support addressing students’ perceived challenges and worries. A good collaboration is also needed to create alternative learning activities during similar situations in the future.

One limitation of the study may be the use of online video conferencing as physical meetings may have given possibilities to probe for a deeper understanding of the students’ perceptions. However, others have found that video conferencing may facilitate focus group discussions and allows participants to contribute to each other’s comments [28]. The strengths of the study are that students at different levels of education with experiences from a variety of clinical placements participated. Still, the results mirror experiences limited to these students’ experiences and contexts. Relating the results to learning theories may increase the possibilities of transfer to other contexts.

## Conclusion

Unpredictable situations such as a pandemic can lead to unique learning since “the extraordinary makes the ordinary visible”. This study shows that students learn things additional to the formal learning outcomes and that the experiences strengthen their will to become nurses. Challenges due to a crisis can become important driving forces for learning, if not experienced as overwhelming. Some students felt they received space for their own initiatives and responsibility while others felt lost and abandoned. Students might need support from supervisors and teachers to take on the challenges and turn it into learning. The students’ worries should be addressed both at the clinical placement and the university, and a good collaboration is needed. Preparing for a crisis means preparing for an unknown future. In addition to epistemological knowledge the students also need ontological challenges. This can be accomplished by letting students experience dilemmas and uncertain situations in a safe environment, like in simulation or role-play. Future research is suggested to explore how such learning activities affect students’ learning and preparedness for the unknown future.

## Data Availability

The datasets generated and analysed during the current study are not publicly available due to ethical reasons connected to the students’ informed consent. The data generated during this study consist of transcribed audio recordings of participants who have been guaranteed confidential handling of data. On reasonable request, data can be made available from the corresponding author.
